# Comparison of cervical disc arthroplasty and anterior cervical discectomy and fusion for the treatment of cervical disc degenerative diseases on the basis of more than 60 months of follow-up: a systematic review and meta-analysis

**DOI:** 10.1186/s12883-020-01717-0

**Published:** 2020-04-20

**Authors:** Yijian Zhang, Nanning Lv, Fan He, Bin Pi, Hao Liu, Angela Carley Chen, Huilin Yang, Mingming Liu, Xuesong Zhu

**Affiliations:** 1 Department of Orthopedics, The First Affiliated Hospital of Soochow University, 899, Pinghai Road, Suzhou, 215006 China; 2 Orthopedic Institute, Soochow University, Suzhou, 215006 China; 3 Department of Orthopedic Surgery, The Second People’s Hospital of Lianyungang, Lianyungang, 222003 Jiangsu China; 4 School of Public Health and Health Systems, University of Waterloo, Waterloo, Ontario N2L 3G1 Canada

**Keywords:** Cervical disc arthroplasty, Anterior cervical discectomy and fusion, Cervical degenerative disc diseases, Long-term follow-up

## Abstract

**Background:**

This meta-analysis was designed to investigate the long-term efficacy and safety between cervical disc arthroplasty (CDA) and anterior cervical discectomy and fusion (ACDF) in treating cervical disc degenerative diseases (CDDDs).

**Methods:**

Literature search was performed on Pubmed, Embase, Cochrane Library, and Web of Science before Jan 2019. Surgical details, clinical outcomes, range of motion (ROM), complications, and reoperation rates between CDA and ACDF groups were compared and analyzed. A fixed- or random-effects model was applied based on different heterogeneity. STATA (Version 11.0) software was used to perform data analysis.

**Results:**

A total of 13 randomized controlled trial studies with more than 60 months of follow-up (mean 83.1 months) were enrolled in this meta-analysis. Pool results indicated that the CDA group exhibited significantly better outcomes in clinical scores (odds ratio [OR] = 1.54, 95% confidence interval [CI]: 1.15–2.08, *p* = 0.004) and preservation of ROM (mean difference = 1.77, 95% CI: 1.60–1.95, *p* < 0.001) than the ACDF group. Meanwhile, the incidence of adjacent segment disease (ASD) (OR = 0.51, 95% CI: 0.35–0.76, *p* = 0.001) and occurrence of reoperation (OR = 0.41, 95% CI: 0.25–0.69, *p* = 0.001) were lower in the CDA group than in the ACDF group.

**Conclusions:**

At long-term follow-up, CDA showed better efficacy in terms of clinical outcomes, ROM, ASD, and reoperation than ACDF for treating CDDDs. However, our results require further validation in large-sample and high-quality studies.

## Background

In the past several decades, anterior cervical discectomy and fusion (ACDF) has been applied to multiple cervical disorders, including cervical spondylotic myelopathy, cervical spondylotic radiculopathy, and cervical ossification of posterior longitudinal ligament, for its satisfactory clinical efficacy [1]. In the ACDF procedure, directed decompression of the nucleus pulposus and osteophyte can be performed under clear vision during operation [2]. Meanwhile, the physical sagittal alignment of cervical spine can be restored with the inserted cage in the intervertebral space, fixed screws, and anterior plates [3]. However, a solid bony fusion in this procedure can change the range of motion (ROM) and mechanical load of adjacent segments, which can cause subsequent adjacent segment disease (ASD) [4].

Therefore, to decrease the risk of ASD in cervical surgery, cervical disc arthroplasty (CDA) was introduced as an alternative treatment for cervical disc degenerative diseases (CDDDs) in the past 20 years [5]. With a mobile device between two contiguous vertebrae, the mobility of operated segments can be preserved, which may potentially decrease the incidence of ASD postoperatively [6]. Moreover, CDA was reported to show better improvement in clinical functions than fusion surgery because the normal kinematics of the involved segments are maintained [7]. Numerous studies have compared the clinical results and complications between CDA and ACDF, but the conclusions are inconsistent [8, 9].

Though several meta-analyses have compared CDA and ACDF, most of them are based on short-term follow-ups [10, 11]. To the best of our knowledge, few studies have examined the long-term efficacy between the two procedures. Hence, in the present study, we performed a systematic review and meta-analysis to investigate the long-term radiographic data, clinical outcomes, and other complications between CDA and ACDF for the treatment of CDDDs.

## Methods

### Literature search

The present meta-analysis was based on the guidelines listed in the Preferred Reporting Items for Systematic Reviews and Meta-Analysis statement [12]. Electronic search was performed on PubMed, Embase, Scopus, Cochrane Database, and Web of Science from the dates of inception to January 2019. In the search strategy, the following controlled vocabulary (Emtree of Embase and MeSH of PubMed) and keywords were used: (“cervical disc arthroplasty” OR “cervical disc replacement” OR “CDA” OR “CDR”) AND (“anterior cervical discectomy and fusion” OR “ACDF”). The reference lists of the enrolled studies were searched for missed eligible studies. The literature search was restricted to the English language.

### Selection criteria

The inclusion criteria for identification of studies were as follows: (1) randomized controlled trials (RCTs), (2) comparison between two surgical procedures (CDA and ACDF), (3) follow-up time of more than 60 months, and (4) reporting at least one surgical related outcome. The studies that satisfied the following criteria were eliminated: (1) lack of comparative data, (2) insufficient follow-up, and (3) biomechanical or in vitro studies.

### Assessment of quality

To evaluate the quality of evidence of the identified studies, the Cochrane Collaboration tool was used to assess the RCTs [13]. Each identified trial was reviewed and scored as high, low, and unclear risks according to selection, performance, detection, attribution, reporting, and other potential biases. Two reviewers (YJ Z and B P) reviewed and assessed each study independently, and a third reviewer (HL Y) was consulted to solve disagreements.

### Data extraction

The data extracted from the enrolled studies for synthesis and analysis included authors, date of publication, study design, study country, patient characteristics (distribution of sex and age), surgical prosthesis, follow-up, neurological success, overall success, neck disability index (NDI), short-form questionnaire for physical health (SF-PCS), arm pain, neck pain, ROM, ASD, adverse events, reoperation at index level, and reoperation at adjacent level.

### Statistical analysis

Meta-analysis was performed using the STATA (Version 11.0) software. For binary variables, the odds ratio (OR) was used for evaluation, while mean difference (MD) was applied for continuous variables. The heterogeneity of studies was estimated using the *I*^*2*^ tests: low heterogeneity (*I*^*2*^ < 30%), moderate heterogeneity (30% < *I*^*2*^ < 50%), and substantial heterogeneity (*I*^*2*^ > 50%). The fixed-effects model was adopted when *I*^*2*^ < 50%, and the random-effects model was applied when *I*^*2*^ > 50%. The minimal clinically important difference (MCID) for NDI, SF-PCS, arm pain and neck pain was set as 2.5 according to previous studies [14, 15]. Publication bias was assessed by Egger’s test, and sensitivity analysis was used to confirm the stability of results. A two-tail *p*-value of less than 0.05 was considered significant.

## Results

### Search results

**Fig. 1:**
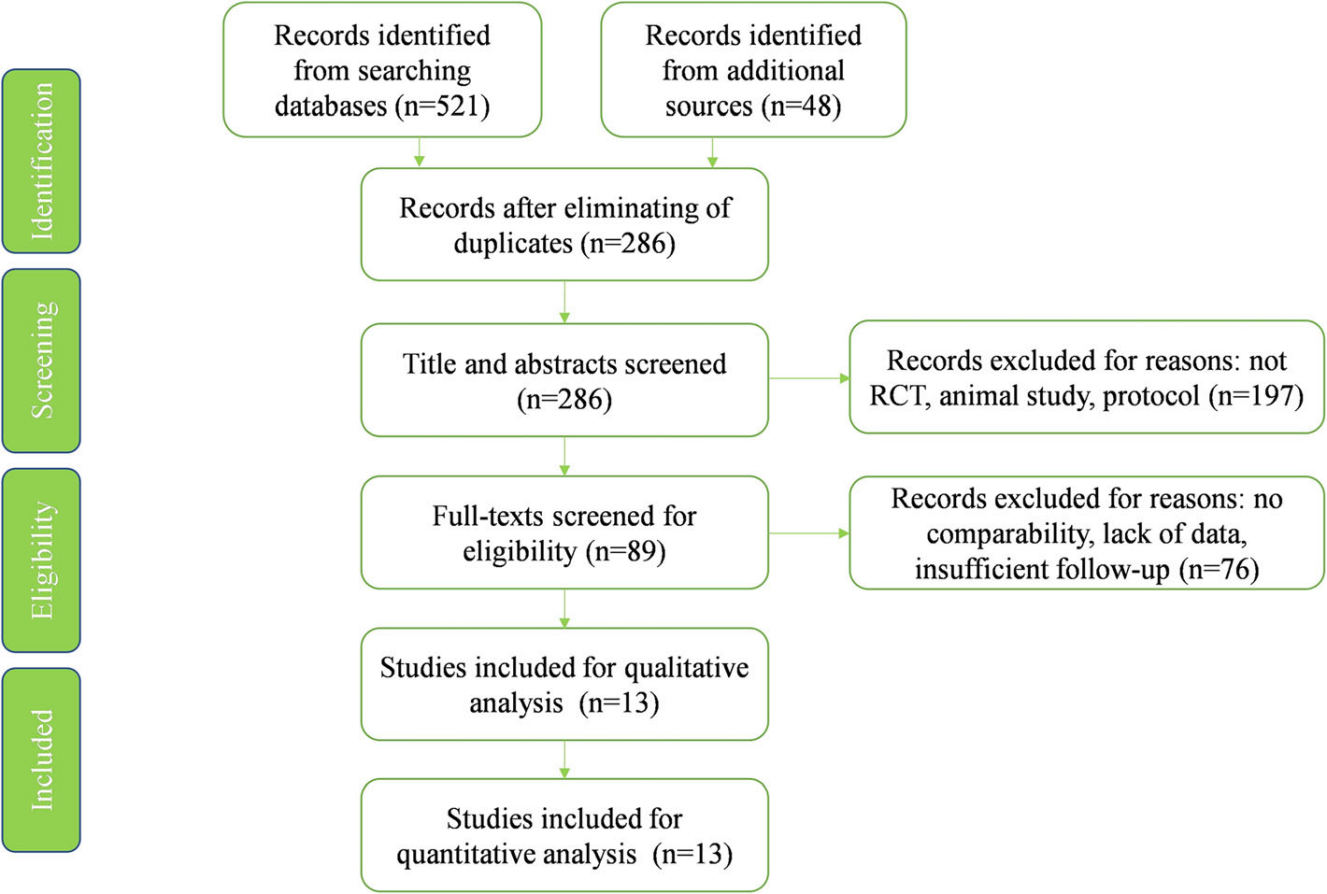
Study flow diagram of literature search

A total of 569 studies were searched at the initial searching process. After eliminating of duplicate studies, 286 studies were screened based on titles and abstracts. The 89 potential eligible studies that remained were full-text reviewed. Ultimately, 13 articles were enrolled in this meta-analysis for qualitative and quantitative analyses (Fig. 1).

### Demographic data

Thirteen RCT studies published between 2013 and 2018 were included in the present study. The distributions of prosthesis in CDA were Bryan (3 studies), Bryan and KineflexC (1 study), Prestige (1 study), Prestige LP (2 study), ProDisc-C (2 studies), Mobi-C (3 studies), and PCM (1 study). The mean follow-up of the enrolled studies was 83.1 months, ranging from 60 months to 120 months. The specific characteristics of all included studies are listed in Table 1.

**Table 1:** Characteristics of 13 enrolled RCT studies

Authors	Year	CDA (N)	CDA (Age)	CDA (Male %)	ACDF (N)	ACDF (Age)	ACDF (Male %)	Prothesis	Follow-up
Burkus et al.	2014	276	43.3	46.4	265	43.9	46.0	Prestige	84 months
Coric et al.	2013	41	49.5	39.0	33	49.3	42.4	Bryan and KineflexC	72 months
Ghobrial et al.	2018	242	Not provided	Not provided	221	Not provided	Not provided	Bryan	120 months
Gornet et al.	2016	280	44.5 ± 8.8	46.1	265	43.9 ± 8.8	46	Prestige LP	84 months
Hisey et al.	2016	164	43.3 ± 9.2	47.6	81	44.0 ± 8.2	44.4	Mobi-C	60 months
Jackson et al.	2016	413	Not provided	Not provided	186	Not provided	Not provided	Mobi-C	60 months
Janssen et al.	2015	103	42.1 ± 8.4	45	106	43.5 ± 7.2	46	ProDisc-C	84 months
Lanman et al.	2017	209	47.1 ± 8.3	44	188	47.3 ± 7.7	47.9	Prestige LP	84 months
Loimeau et al.	2016	22	Not provided	Not provided	22	Not provided	Not provided	ProDisc-C	84 months
Miller et al.	2018	34	Not provided	Not provided	36	Not provided	Not provided	Bryan	84 months
Philips et al.	2015	224	Not provided	Not provided	192	Not provided	Not provided	PCM	60 months
Radcliff et al.	2017	389	44.5 ± 8.6	49	186	45.2 ± 8.1	43.5	Mobi-C	84 months
Sasso et al.	2016	22	Not provided	Not provided	25	Not provided	Not provided	Bryan	120 months

### Quality assessment

**Fig. 2:**
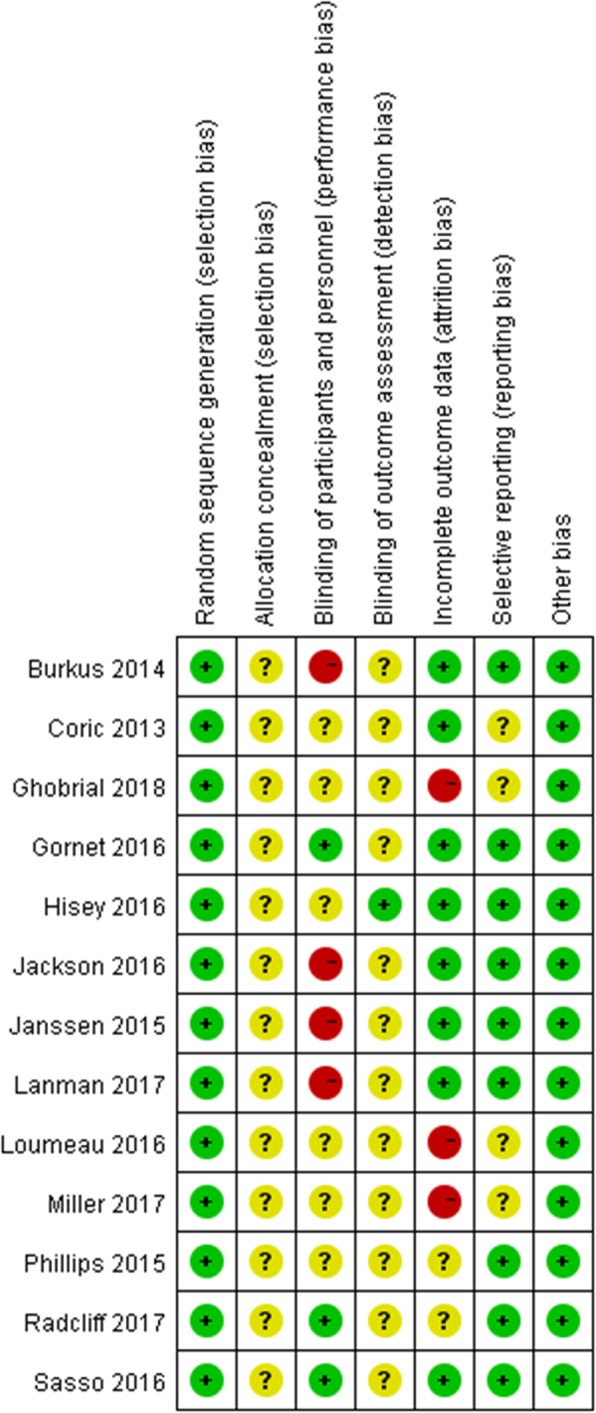
Assessment of risk of bias for RCT: “+”: low risk of bias; “−”: high risk of bias; “?”: unclear risk of bias

The majority of the 13 eligible studies were well designed and of high quality. All studies were rated as “low risk of bias” according to the Cochrane Handbook for Systematic Review of Interventions (Fig. 2).

### Meta-analysis outcome

**Fig. 3:**
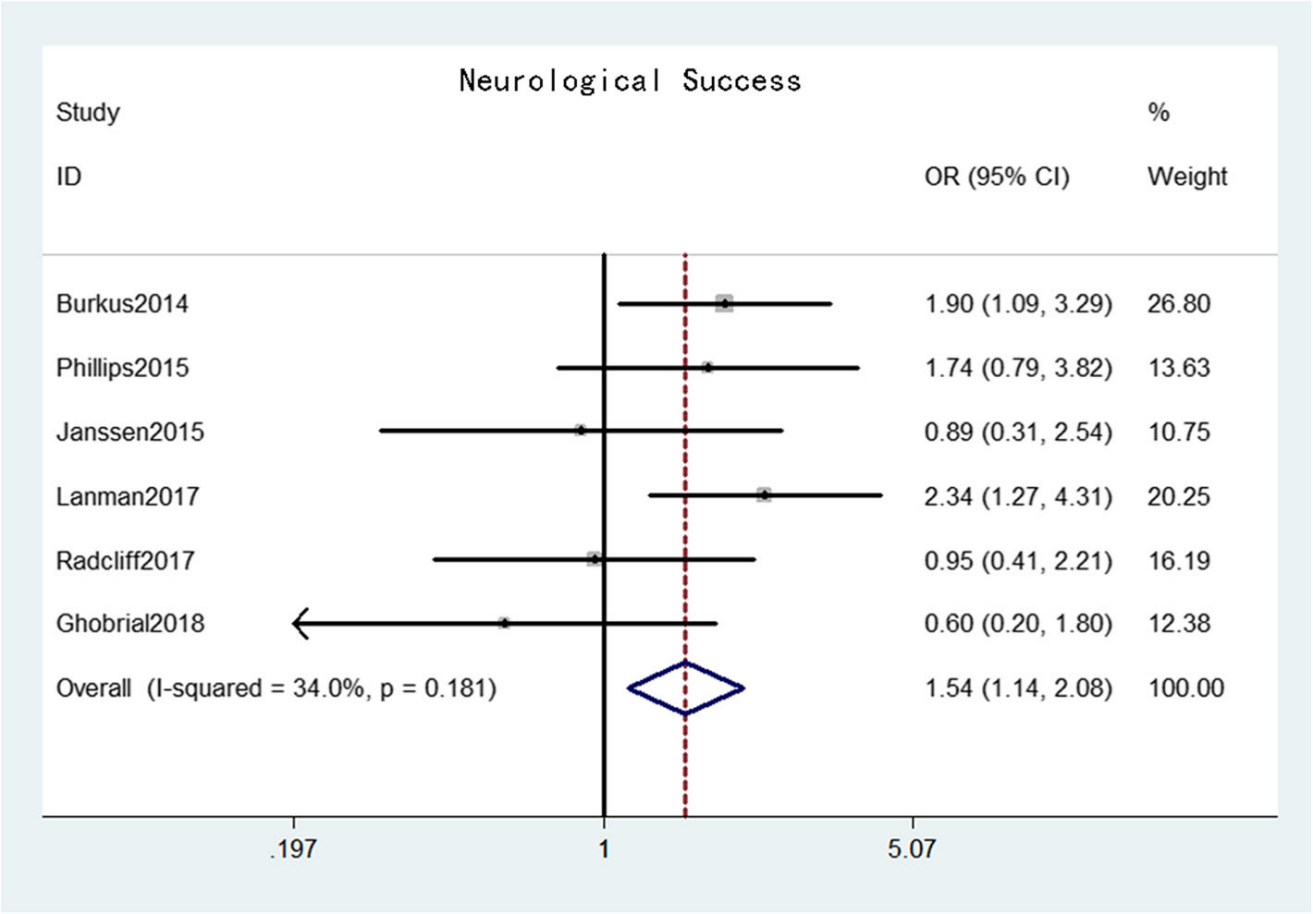
Forest plot of neurological success between CDA and ACDF groups

**Fig. 4:**
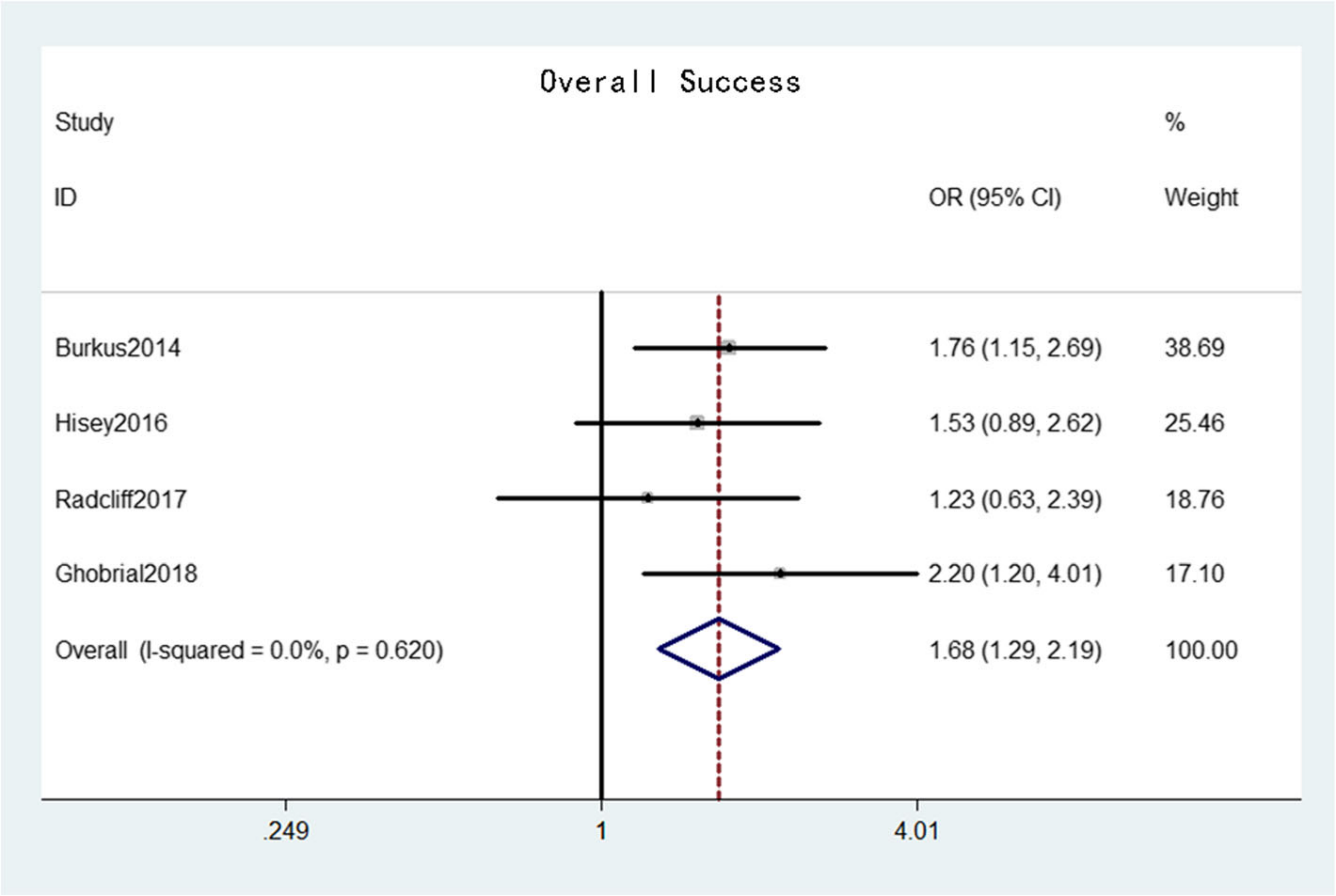
Forest plot of overall success between CDA and ACDF groups

Neurological success was reported in six studies [16–21] that comprised 944 patients in the CDA group and 747 patients in the ACDF group. The pooled results indicated that the neurological success rate in the CDA group was significantly higher than that in the ACDF group (OR = 1.54, 95% confidence interval [CI]: 1.14–2.08, *p* = 0.004) with moderate heterogeneity (*I*^*2*^ = 34.0%, *p* = 0.18) (Fig. 3). Overall success was reported in four studies [16, 20–22] that comprised 620 patients in the CDA group and 418 patients in the ACDF group. The pooled results indicated that the overall success rate in the CDA group was also significantly higher than that in the ACDF group (OR = 1.68, 95% CI: 1.29–2.19, *p* < 0.001) with low heterogeneity (*I*^*2*^ = 0%, *p* = 0.62) (Fig. 4).

**Fig. 5:**
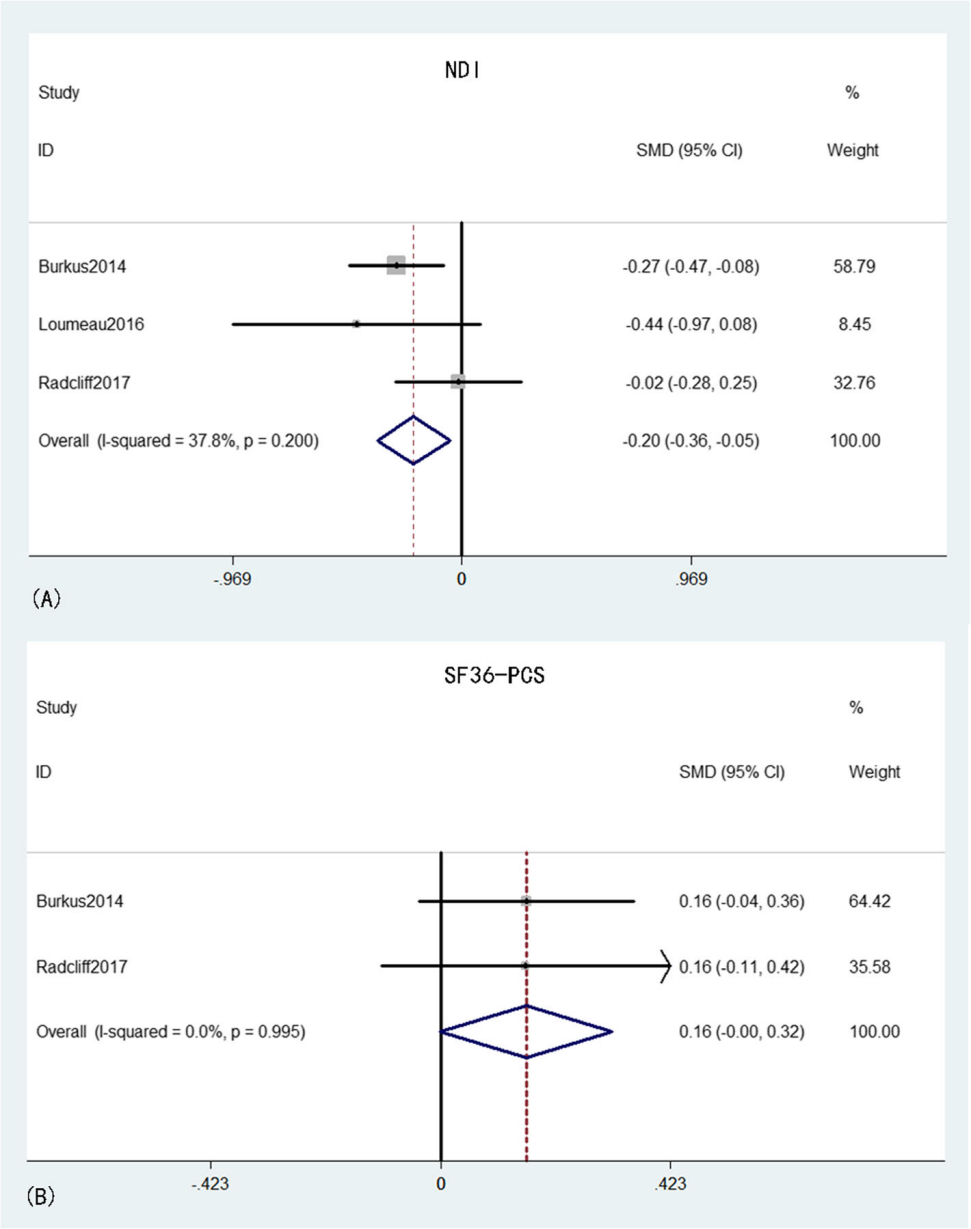
Forest plot of NDI (A) and SF-36 PCS (B) between CDA and ACDF groups

NDI was provided in three studies [16, 20, 23] that comprised 398 patients in the CDA group and 305 patients in the ACDF group. The pooled results indicated that the NDI in the CDA group was significantly lower than that in the ACDF group (MD = − 0.20, 95% CI: − 0.36 to − 0.05, *p* = 0.009), with moderate heterogeneity (*I*^*2*^ = 37.8%, *p* = 0.20) while the effective size of NDI did not exceed the MCID. SF-PCS was obtained from two studies [16, 20] that comprised 376 patients in the CDA group and 264 patients in the ACDF group. The pooled results indicated that the SF-PCS scores were higher in the CDA group than in the ACDF group, with a clear tendency to significance (MD = 0.16, 95% CI: − 0.00–0.32, *p* = 0.05) and an inconspicuous heterogeneity (*I*^*2*^ = 0%, *p* = 0.995) while the effective size of SF-PCS did not exceed the MCID (Fig. 5).

**Fig. 6:**
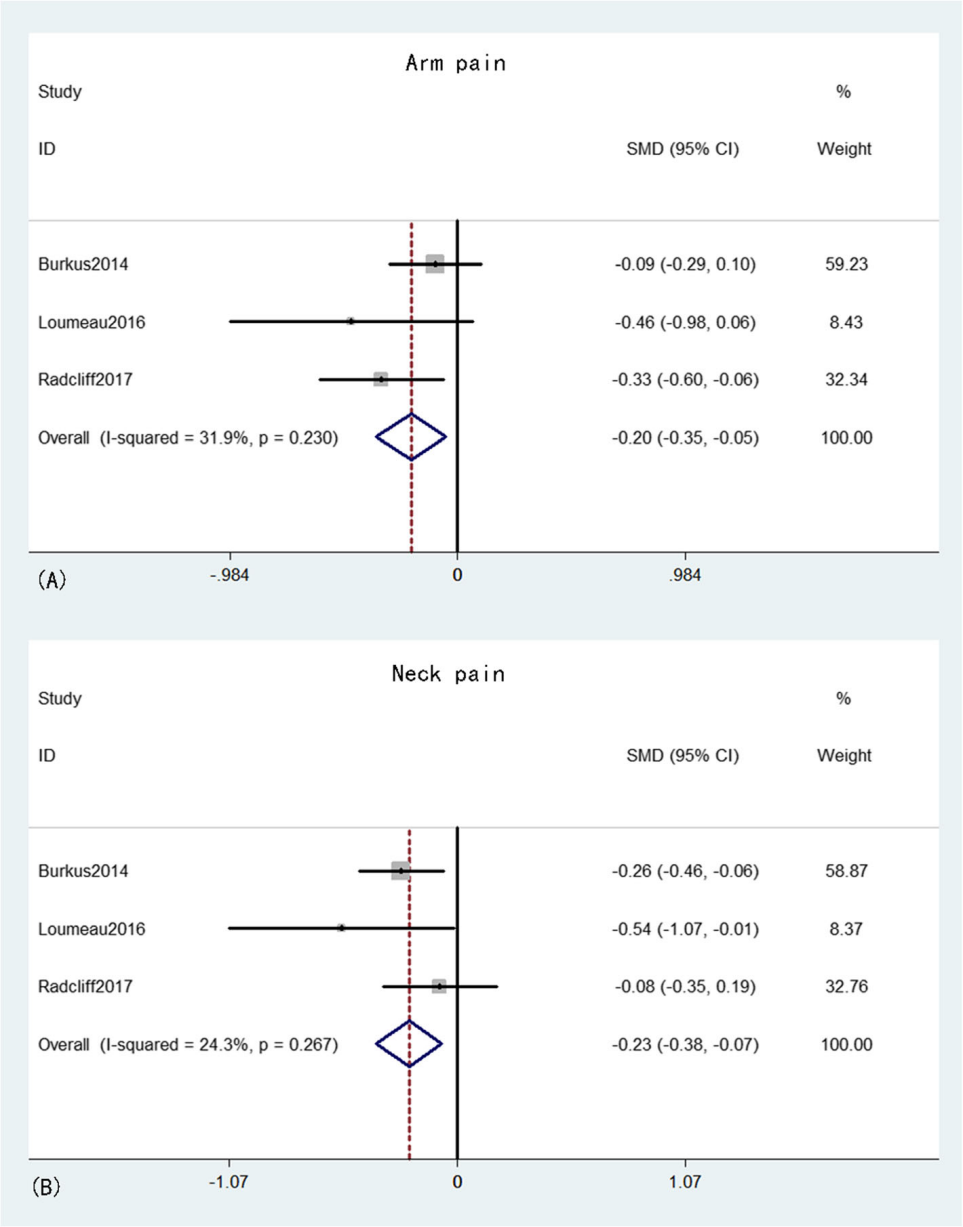
Forest plot of arm pain (A) and neck pain (B) between CDA and ACDF groups

Arm and neck pains were provided in three studies [16, 20, 23] that comprised 398 patients in the CDA group and 305 patients in the ACDF group. The pooled results indicated that both neck and arm pains were significantly better in the CDA group than in the ACDF group (MD = − 0.20, 95% CI: − 0.35 to − 0.05, *p* = 0.01 and MD = − 0.23, 95% CI: − 0.38 to − 0.07, *p* = 0.004, respectively), with inconspicuous heterogeneity (*I*^*2*^ = 31.6%, *p* = 0.23 and *I*^*2*^ = 24.3%, *p* = 0.27, respectively). The effective size of arm and neck pains did not exceed the MCID (Fig. 6).

**Fig. 7:**
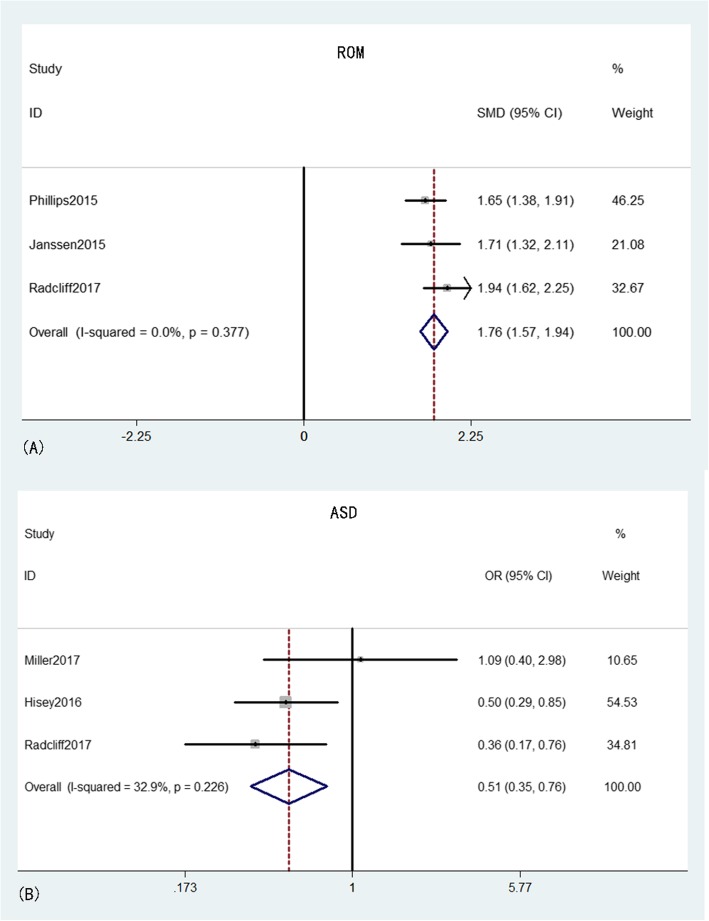
Forest plot of ROM (A) and ASD (B) between CDA and ACDF groups

ROM was reported in three studies [17, 18, 20] that comprised 400 patients in the CDA group and 274 patients in the ACDF group. The pooled results indicated that the ROM in the CDA group was significantly larger than that in the ACDF group (MD = 1.76, 95% CI: 1.57–1.94, *p* < 0.001) with low heterogeneity (*I*^*2*^ = 0%, *p* = 0.38). ASD was provided from three studies [20, 22, 24] that comprised 302 patients in the CDA group and 160 patients in the ACDF group. The pooled results indicated that the ASD rate in the CDA group was significantly lower than that in the ACDF group (OR = 0.51, 95% CI: 0.35–0.76, *p* = 0.001), with moderate heterogeneity (*I*^*2*^ = 32.9%, *p* = 0.23) (Fig. 7).

**Fig. 8:**
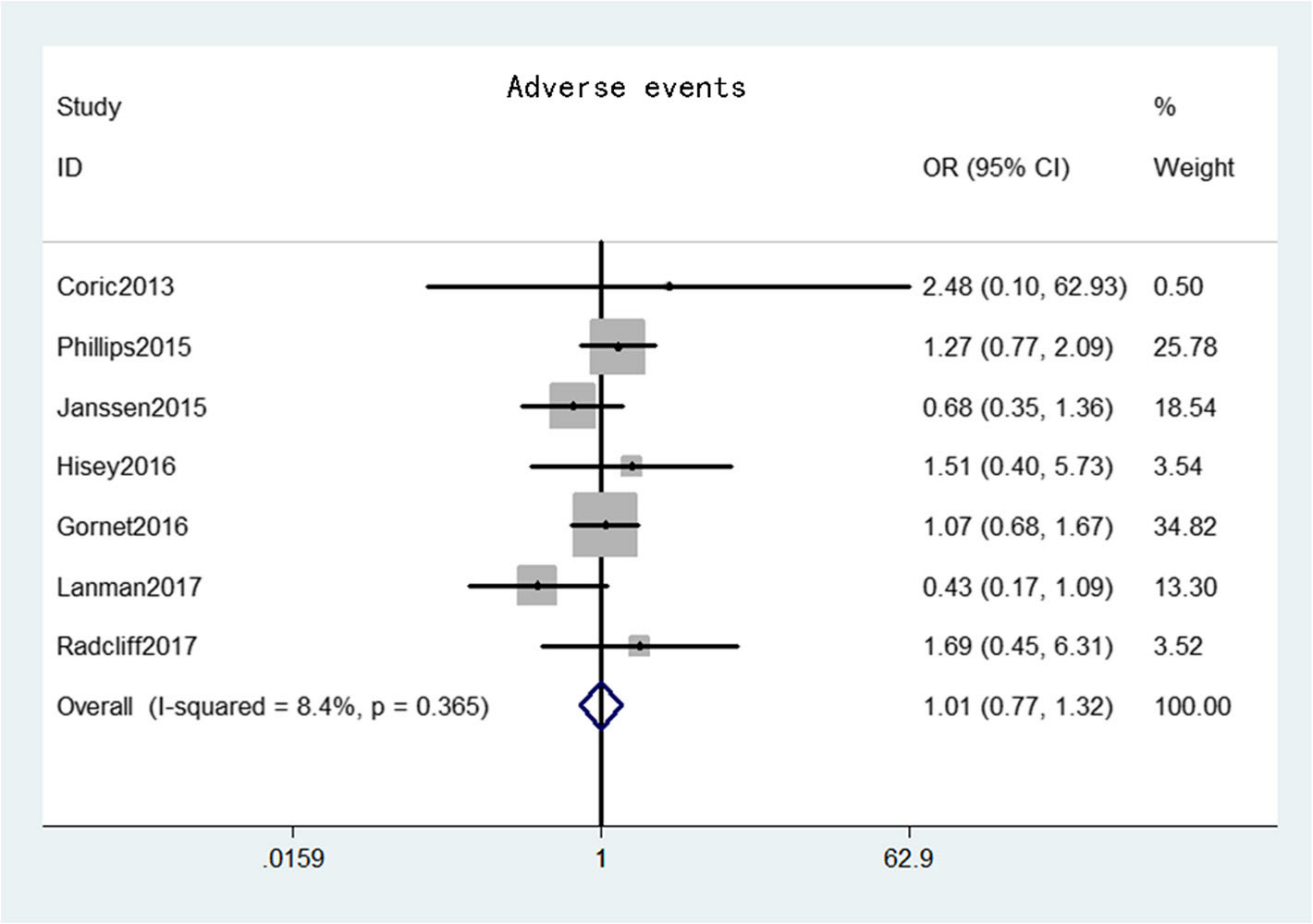
Forest plot of adverse events between CDA and ACDF groups

The adverse event rate was provided from seven studies [17–20, 22, 25, 26] that comprised 1145 patients in the CDA group and 901 patients in the ACDF group. The pooled results indicated the adverse event rate was not significant between the two groups (OR = 1.01, 95% CI: 0.77–1.32, *p* = 0.96), with low heterogeneity (*I*^*2*^ = 8.4%, *p* = 0.37) (Fig. 8).

**Fig. 9:**
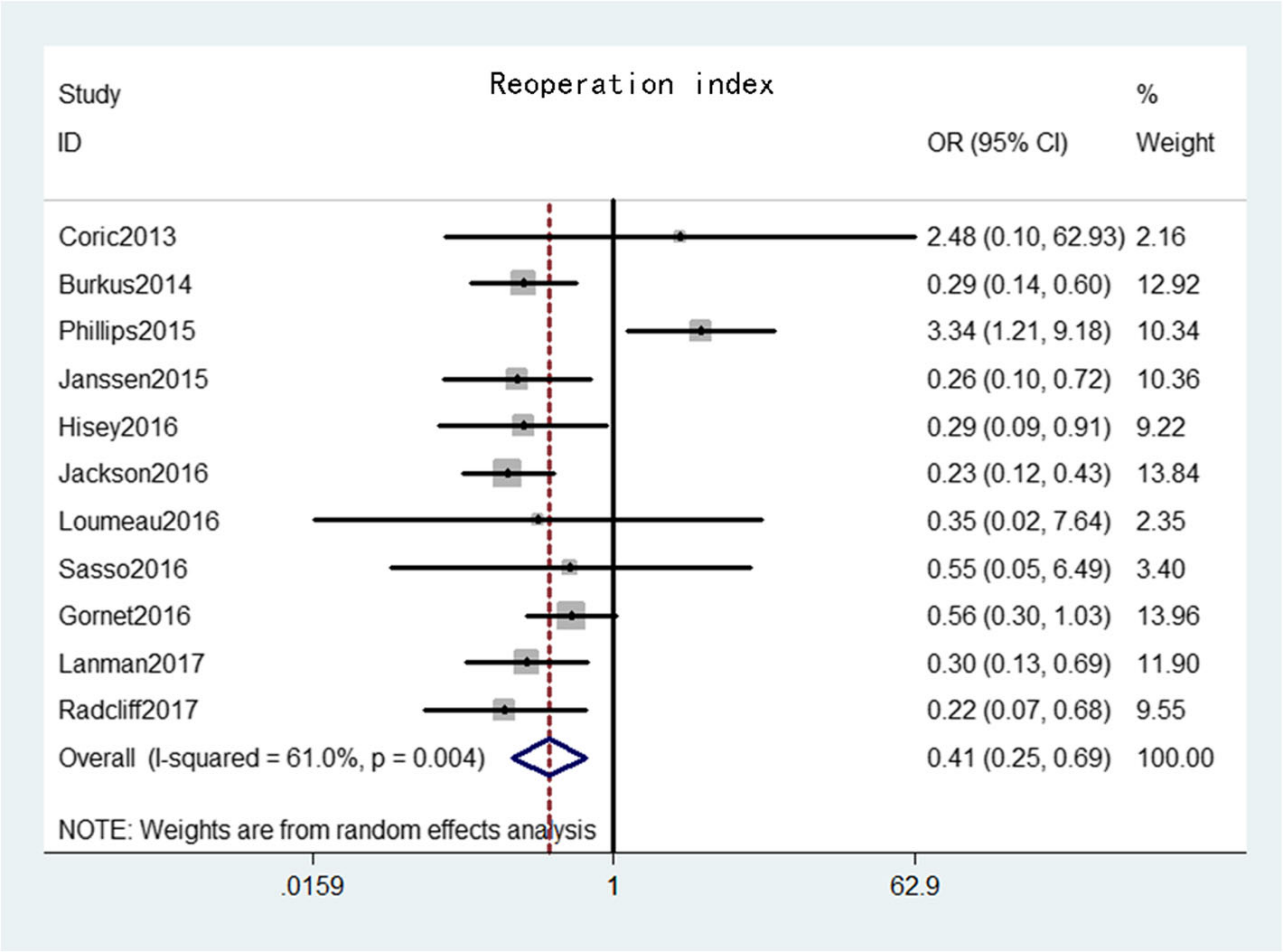
Forest plot of reoperation at index level between CDA and ACDF groups

Reoperation at the index level was provided from 11 studies [16–20, 22, 23, 25–28] that comprised 1811 patients in the CDA group and 1330 patients in the ACDF group. The pooled results indicated that reoperation at the index level rate was significantly lower in the CDA group than in the ACDF group (OR = 0.41, 95% CI: 0.25–0.69, *p* = 0.001), with substantial heterogeneity (*I*^*2*^ = 61.0%, *p* = 0.004) (Fig. 9).

**Fig. 10:**
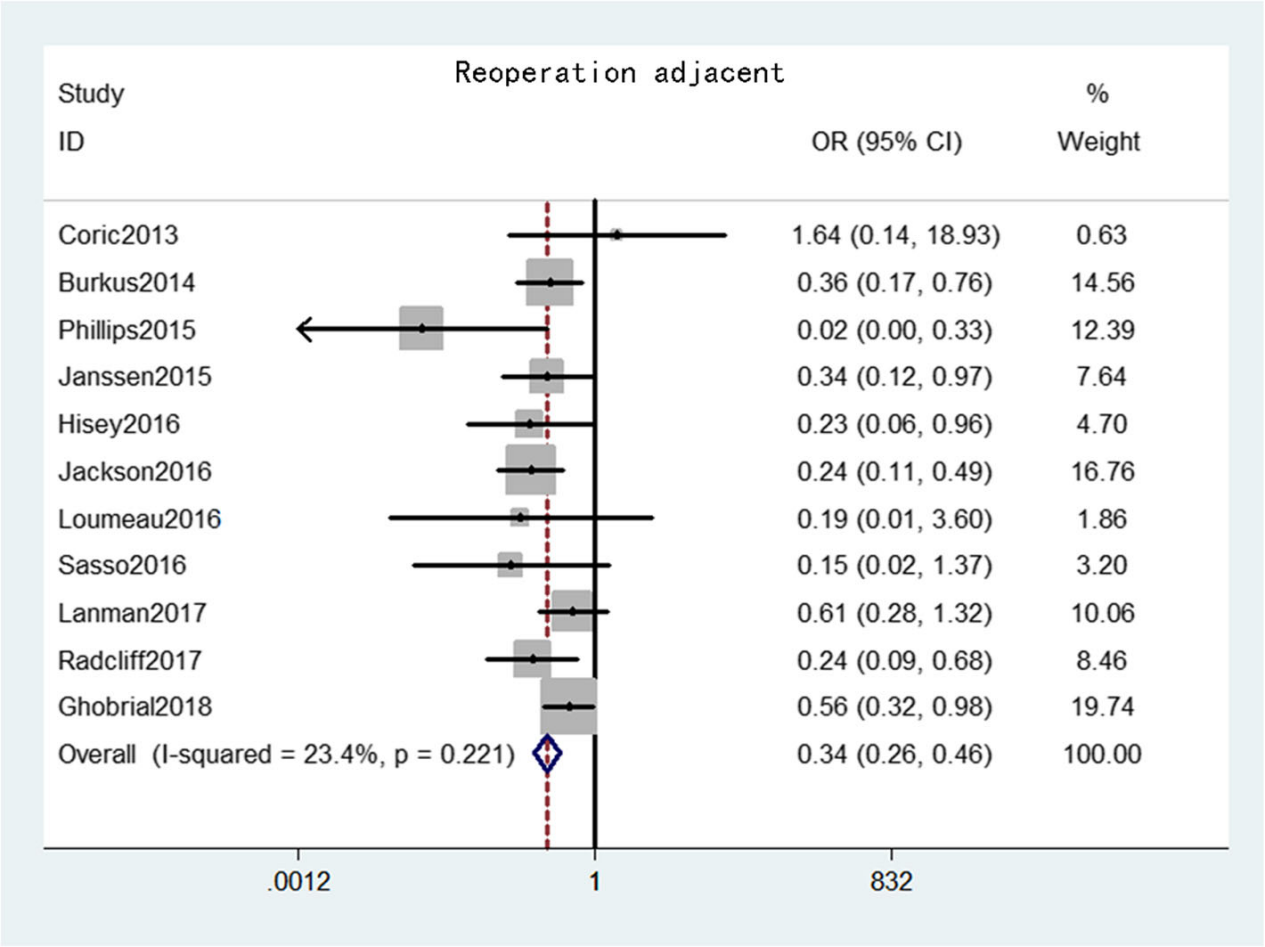
Forest plot of reoperation at adjacent level between CDA and ACDF groups

Reoperation at the adjacent level was provided from 11 studies [16–23, 25, 27, 28] that comprised 1773 patients in the CDA group and 1286 patients in the ACDF group. The pooled results indicated that reoperation at the adjacent level rate was significantly lower in the CDA group than in the ACDF group (OR = 0.34, 95% CI: 0.26–0.46, *p* < 0.001), with low heterogeneity (*I*^*2*^ = 23.4%, *p* = 0.22) (Fig. 10).

### Publication bias

Publication bias was investigated using Begg’s test, and Begg’s funnel plot was constructed to evaluate the publication bias of the included studies. No studies showed obvious asymmetry on two sides, indicating a low publication bias in the present study (Supplement 1).

### Sensitivity analysis

Sensitivity analysis was performed to examine whether removing each study would make a significant change on the overall trend. No altered results were observed after each study was eliminated, suggesting the reliability and stability of results in this meta-analysis (Supplement 2).

## Discussion

ACDF has been applied as an optimal surgical procedure for treating cervical degenerative diseases since it was first reported in 1958 by Robinson and Smith [29]. However, with the widespread use of this procedure, some complications, including decrease of ROM, emergence of ASD, and reoperation, have drawn significant attention. Previous studies reported that the loss of mobility of surgical segments caused by fusion may be a potential reason for ASD and reoperation. By contrast, as a non-fusion decompression method, CDA can preserve the motion of index segments and the natural cervical alignment. ROM, at either operative or adjacent levels, can be maintained effectively after CDA [30]. In recent years, numerous meta-analyses compared the clinical and radiological outcomes between ACDF and CDA to evaluate whether the latter is better at reducing related complications. Luo et al. reported that CDA has significantly lower incidence of ASD and adjacent segment reoperations in cervical degenerative diseases than ACDF [31]. Similar results were also verified in another study, indicating the superior effect of CDA through the fewer adjacent segmental complications [32]. Meanwhile, some studies demonstrated that CDA may show better clinical outcomes and fewer postoperative adverse events than ACDF [33]. Unfortunately, most of them reported short- or mid-term outcomes between the two procedures, while long-term results were still unclear.

In this meta-analysis, the authors conducted a comparison between the surgical successes, clinical outcomes, ROMs, ASDs, and reoperations between CDA and ACDF on the basis of more than 60 months follow-up and obtained meaningful results. For clinical outcomes, at a mean of 83.1 months of follow-up, the NDI, SF-PCS, and neck and arm pains in the CDA group were better than those in the ACDF group, indicating that dynamic implant can achieve better clinical outcomes. However, for the clinical relevance, none of these parameters met the MCID. Therefore, we considered the superior curative outcomes for pain management need more studies to verify. The rates of neurological and overall successes were also higher in the CDA group than in the ACDF group. Both CDA and ACDF can relieve pain symptoms by the adequate decompression of the herniated disc or osteophyte under direct vision through the anterior approach. Meanwhile, compared with ACDF, CDA can preserve the cervical mobility and provide a normal motion pattern of the intact spine, which may be a potential cause of the superior clinical outcomes in the CDA group [34].

As a dynamic device, CDA was designed to preserve the activity of surgical segments, which prevents long-term complications, such as ASD, neck stiffness, and revision. Zeng et al. conducted a seven-year follow-up and observed that the segment motion in the Prestige-LP Disc can be preserved effectively at the final follow-up [35]. Tian et al. performed a comparative study between Bryan disc and anterior fusion surgery, and their results corroborated that the ROM of the former is explicitly better and the mobility can be maintained after 6 years of follow-up [36]. In the present study, as theoretically predicted, the ROM in the CDA group was significantly higher than that in the ACDF group at long-term follow-up, which is consistent with previous studies [17, 18]. Additionally, we observed from the included studies that the ROM can be maintained well and even improved slightly in CDA patients, indicating the satisfactory effect of CDA on the preservation of segmental motion. For patients who demand normal working and mild activity, such maintenance of cervical mobility may benefit the quality of life.

As for complications, though no significant difference in the adverse events was observed between CDA and ACDF. It was worth noting that as a common complication after cervical surgery, the incidence of ASD was seem lower in the CDA group than in the ACDF group with our statistical results, which reveals that CDA may have a potential positive impact on reducing postoperative ASD at long-term follow-up. As mentioned above, due to the loss of segmental motion, patients after ACDF may have abnormal mobility at adjacent levels that can alter the biomechanical load and exacerbate degeneration of adjacent vertebrae [37]. Compared with fusion surgery, the mobility and flexibility of operative levels can be preserved in the CDA group, and a physiological functional spinal unit (FSU) can be conserved, which may benefit the natural degenerative process. Finally, the reoperation rates of the two groups were compared, and CDA exhibited a favorable effect due to the lower reoperation rate, whether at index or adjacent segments [21, 24]. Without doubt, a second operation is another trauma for patients, and prior tissue and scar formation will increase the difficulty and risk of reoperation. Previous studies demonstrated that symptomatic pseudarthrosis is the main cause of reoperation at the treated level, while symptomatic ASD is responsible for reoperation at adjacent levels [38]. Hence, we inferred that the preservation of segmental motion can provide a physical FSU and avoid the generation of pseudarthrosis, which can reduce the reoperation rate at the index level. Meanwhile, a normal segmental mobility after surgery can decrease the excessive motion of adjacent levels and avert the occurrence of ASD, which leads to a low rate of reoperation at adjacent segments.

### Limitation

We considered that the results of the present meta-analysis may be affected by the following reasons: First, the number of included studies was small, which may lead to insufficient evidence. Meanwhile, some included studies were sponsored by companies, which may have introduced some confounding factors. Second, some results have moderate heterogeneity, which may introduce bias. Though we conducted publication bias and sensitivity assessment to consolidate the stability, these results still need to be interpreted with caution. Third, the use of cervical prothesis in different studies was not coincident. Given the limited data, subgroup analysis is difficult to perform according to different devices. Fourth, the reasons for reoperation may sometimes depend on the surgeon’s preference, which will also bring bias to the results. Fifth, though some variables showed significant differences between two groups, the overall effects did not across the MCID. Hence, the clinical reference for these data should be considered prudently. Based on the above limitations, we believe that the combined results of the present study should be accepted carefully, and more high-quality studies with larger samples and detailed data are needed.

## Conclusion

For the treatment of CDDDs, CDA is superior than ACDF in terms of improving clinical outcomes, preserving the ROM, ASD incidence, and reoperation rate at long-term follow-up. However, more high-quality, large-sample, and strong-evidenced studies are needed to verify our results.

## Supplementary information

**Additional file 1.**

**Additional file 2.**

## Data Availability

All data generated or analyzed in this work are included in the published version.

## References

[1] Yudoyono F, Cho PG, Park SH (2018). Factors associated with surgical outcomes of cervical ossification of the posterior longitudinal ligament [J]. Medicine.

[2] Ren J, Li R, Zhu K (2019). Biomechanical comparison of percutaneous posterior endoscopic cervical discectomy and anterior cervical decompression and fusion on the treatment of cervical spondylotic radiculopathy [J]. J Orthop Surg Res.

[3] Zhang D, Liu B, Zhu J (2019). Comparison of clinical and radiologic outcomes between self-locking stand-alone cage and cage with anterior plate for multilevel anterior cervical discectomy and fusion: a meta-analysis [J]. World Neurosurg.

[4] Vleggeert-lankamp CL, Janssen TMH, Van Zwet E (2018). the NECK trial: effectiveness of anterior cervical discectomy with or without interbody fusion and arthroplasty in the treatment of cervical disc herniation; a double-blinded randomized controlled trial [J]. The spine journal : official journal of the North American Spine Society.

[5] Chang CC, Huang WC, Wu JC (2018). The option of motion preservation in cervical Spondylosis: cervical disc Arthroplasty update [J]. Neurospine.

[6] Hill P, Vaishnav A, Kushwaha B (2018). Comparison of inpatient and outpatient preoperative factors and postoperative outcomes in 2-level cervical disc Arthroplasty [J]. Neurospine.

[7] Lei T, Liu Y, Wang H (2016). Clinical and radiological analysis of Bryan cervical disc arthroplasty: eight-year follow-up results compared with anterior cervical discectomy and fusion [J]. Int Orthop.

[8] Sasso RC, Anderson PA, Riew KD (2011). Results of cervical arthroplasty compared with anterior discectomy and fusion: four-year clinical outcomes in a prospective, randomized controlled trial [J]. J Bone Joint Surg Am.

[9] Grasso G. Clinical and radiological features of hybrid surgery in multilevel cervical degenerative disc disease [J]. European spine journal : official publication of the European Spine Society, the European Spinal Deformity Society, and the European Section of the Cervical Spine Research Society, 2015, 24 Suppl 7(842–848). 10.1007/s00586-015-4281-7 26463866

[10] Shangguan L, Ning GZ, Tang Y (2017). Discover cervical disc arthroplasty versus anterior cervical discectomy and fusion in symptomatic cervical disc diseases: a meta-analysis [J]. PLoS One.

[11] Zou S, Gao J, Xu B (2017). Anterior cervical discectomy and fusion (ACDF) versus cervical disc arthroplasty (CDA) for two contiguous levels cervical disc degenerative disease: a meta-analysis of randomized controlled trials [J]. European spine journal : official publication of the European Spine Society, the European Spinal Deformity Society, and the European Section of the Cervical Spine Research Society.

[12] Page MJ, Moher D (2017). Evaluations of the uptake and impact of the Preferred Reporting Items for Systematic reviews and Meta-Analyses (PRISMA) Statement and extensions: a scoping review [J]. Systematic reviews.

[13] Higgins JP, Altman DG, Gotzsche PC (2011). The Cochrane Collaboration's tool for assessing risk of bias in randomised trials [J]. Bmj.

[14] Angst F, Aeschlimann A, Stucki G (2001). Smallest detectable and minimal clinically important differences of rehabilitation intervention with their implications for required sample sizes using WOMAC and SF-36 quality of life measurement instruments in patients with osteoarthritis of the lower extremities [J]. Arthritis Rheum.

[15] Katz NP, Paillard FC, Ekman E (2015). Determining the clinical importance of treatment benefits for interventions for painful orthopedic conditions [J]. J Orthop Surg Res.

[16] Burkus JK, Traynelis VC, Haid RW (2014). Clinical and radiographic analysis of an artificial cervical disc: 7-year follow-up from the prestige prospective randomized controlled clinical trial: clinical article [J]. J Neurosurg Spine.

[17] Janssen ME, Zigler JE, Spivak JM (2015). ProDisc-C Total disc replacement versus anterior cervical discectomy and fusion for single-level symptomatic cervical disc disease: seven-year follow-up of the prospective randomized U.S. Food and Drug Administration investigational device exemption study [J]. J Bone Joint Surg Am.

[18] Phillips FM, Geisler FH, Gilder KM (2015). Long-term outcomes of the US FDA IDE prospective, randomized controlled clinical trial comparing PCM cervical disc Arthroplasty with anterior cervical discectomy and fusion [J]. Spine.

[19] Lanman TH, Burkus JK, Dryer RG (2017). Long-term clinical and radiographic outcomes of the prestige LP artificial cervical disc replacement at 2 levels: results from a prospective randomized controlled clinical trial [J]. J Neurosurg Spine.

[20] Radcliff K, Davis RJ, Hisey MS (2017). Long-term Evaluation of Cervical Disc Arthroplasty with the Mobi-C(c) Cervical Disc: A Randomized, Prospective, Multicenter Clinical Trial with Seven-Year Follow-up [J]. International journal of spine surgery.

[21] Ghobrial GM, Lavelle WF, Florman JE (2019). Symptomatic adjacent level disease requiring surgery: analysis of 10-year results from a prospective, randomized, clinical trial comparing cervical disc Arthroplasty to anterior cervical fusion [J]. Neurosurgery.

[22] Hisey MS, Zigler JE, Jackson R (2016). Prospective, Randomized Comparison of One-level Mobi-C Cervical Total Disc Replacement vs. Anterior Cervical Discectomy and Fusion: Results at 5-year Follow-up [J]. International journal of spine surgery.

[23] Loumeau TP, Darden BV, Kesman TJ (2016). A RCT comparing 7-year clinical outcomes of one level symptomatic cervical disc disease (SCDD) following ProDisc-C total disc arthroplasty (TDA) versus anterior cervical discectomy and fusion (ACDF) [J]. European spine journal : official publication of the European Spine Society, the European Spinal Deformity Society, and the European Section of the Cervical Spine Research Society.

[24] Miller J, Sasso R, Anderson P (2018). Adjacent level degeneration: Bryan Total disc Arthroplasty versus anterior cervical discectomy and fusion [J]. Clinical spine surgery.

[25] Coric D, Kim PK, Clemente JD (2013). Prospective randomized study of cervical arthroplasty and anterior cervical discectomy and fusion with long-term follow-up: results in 74 patients from a single site [J]. J Neurosurg Spine.

[26] Gornet MF, Burkus JK, Shaffrey ME (2016). Cervical Disc Arthroplasty with Prestige LP Disc Versus Anterior Cervical Discectomy and Fusion: Seven-Year Outcomes [J]. International journal of spine surgery.

[27] Jackson RJ, Davis RJ, Hoffman GA (2016). Subsequent surgery rates after cervical total disc replacement using a Mobi-C cervical disc prosthesis versus anterior cervical discectomy and fusion: a prospective randomized clinical trial with 5-year follow-up [J]. J Neurosurg Spine.

[28] Sasso WR, Smucker JD, Sasso MP (2017). Long-term clinical outcomes of cervical disc Arthroplasty: a prospective, randomized, controlled trial [J]. Spine.

[29] Burkhardt BW, Brielmaier M, Schwerdtfeger K (2017). Smith-Robinson procedure with and without Caspar plating as a treatment for cervical spondylotic myelopathy: a 26-year follow-up of 23 patients [J]. European spine journal : official publication of the European Spine Society, the European Spinal Deformity Society, and the European Section of the Cervical Spine Research Society.

[30] Song Q, He D, Han X (2018). Clinical and radiological outcomes of cervical disc arthroplasty: ten year follow-up study [J]. Int Orthop.

[31] Luo J, Wang H, Peng J (2018). Rate of Adjacent Segment Degeneration of Cervical Disc Arthroplasty Versus Fusion Meta-Analysis of Randomized Controlled Trials [J]. World Neurosurg.

[32] Dong L, Xu Z, Chen X (2017). The change of adjacent segment after cervical disc arthroplasty compared with anterior cervical discectomy and fusion: a meta-analysis of randomized controlled trials [J]. The spine journal : official journal of the North American Spine Society.

[33] Hu Y, Lv G, Ren S (2016). Mid- to long-term outcomes of cervical disc Arthroplasty versus anterior cervical discectomy and fusion for treatment of symptomatic cervical disc disease: a systematic review and meta-analysis of eight prospective randomized controlled trials [J]. PLoS One.

[34] Rao MJ, Nie SP, Xiao BW (2015). Cervical disc arthroplasty versus anterior cervical discectomy and fusion for treatment of symptomatic cervical disc disease: a meta-analysis of randomized controlled trials [J]. Arch Orthop Trauma Surg.

[35] Zeng J, Liu H, Wang B (2018). Clinical and radiographic comparison of cervical disc arthroplasty with Prestige-LP Disc and anterior cervical fusion: A minimum 6-year follow-up study [J]. Clin Neurol Neurosurg.

[36] Tian W, Yan K, Han X (2017). Comparison of the clinical and radiographic results between cervical artificial disk replacement and anterior cervical fusion: a 6-year prospective nonrandomized comparative study [J]. Clinical spine surgery.

[37] Zhao H, Duan LJ, Gao YS (2018). What is the superior surgical strategy for bi-level cervical spondylosis-anterior cervical disc replacement or anterior cervical decompression and fusion?: a meta-analysis from 11 studies [J]. Medicine.

[38] Delamarter RB, Zigler J (2013). Five-year reoperation rates, cervical total disc replacement versus fusion, results of a prospective randomized clinical trial [J]. Spine.

