# Supplementary figures and images for: Comparison of cervical disc arthroplasty and anterior cervical discectomy and fusion for the treatment of cervical disc degenerative diseases on the basis of more than 60 months of follow-up: a systematic review and meta-analysis
Source: BMC Neurol. 2020 Apr 20;20:143. doi: 10.1186/s12883-020-01717-0 (PMC7171870; doi:10.1186/s12883-020-01717-0)

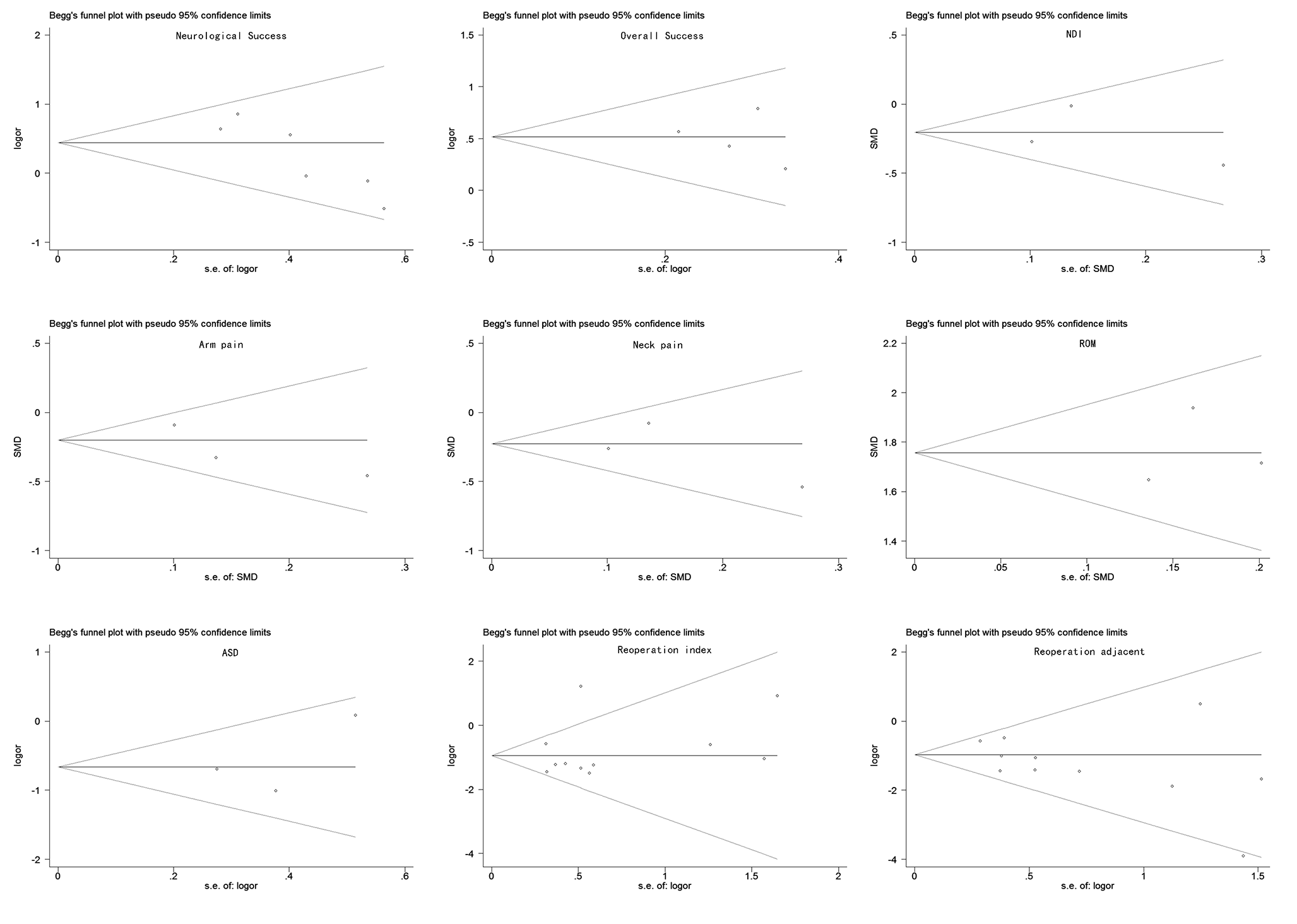

Supplement: Supplementary file 1 — Additional file 1. [file 12883_2020_1717_MOESM1_ESM.tif]

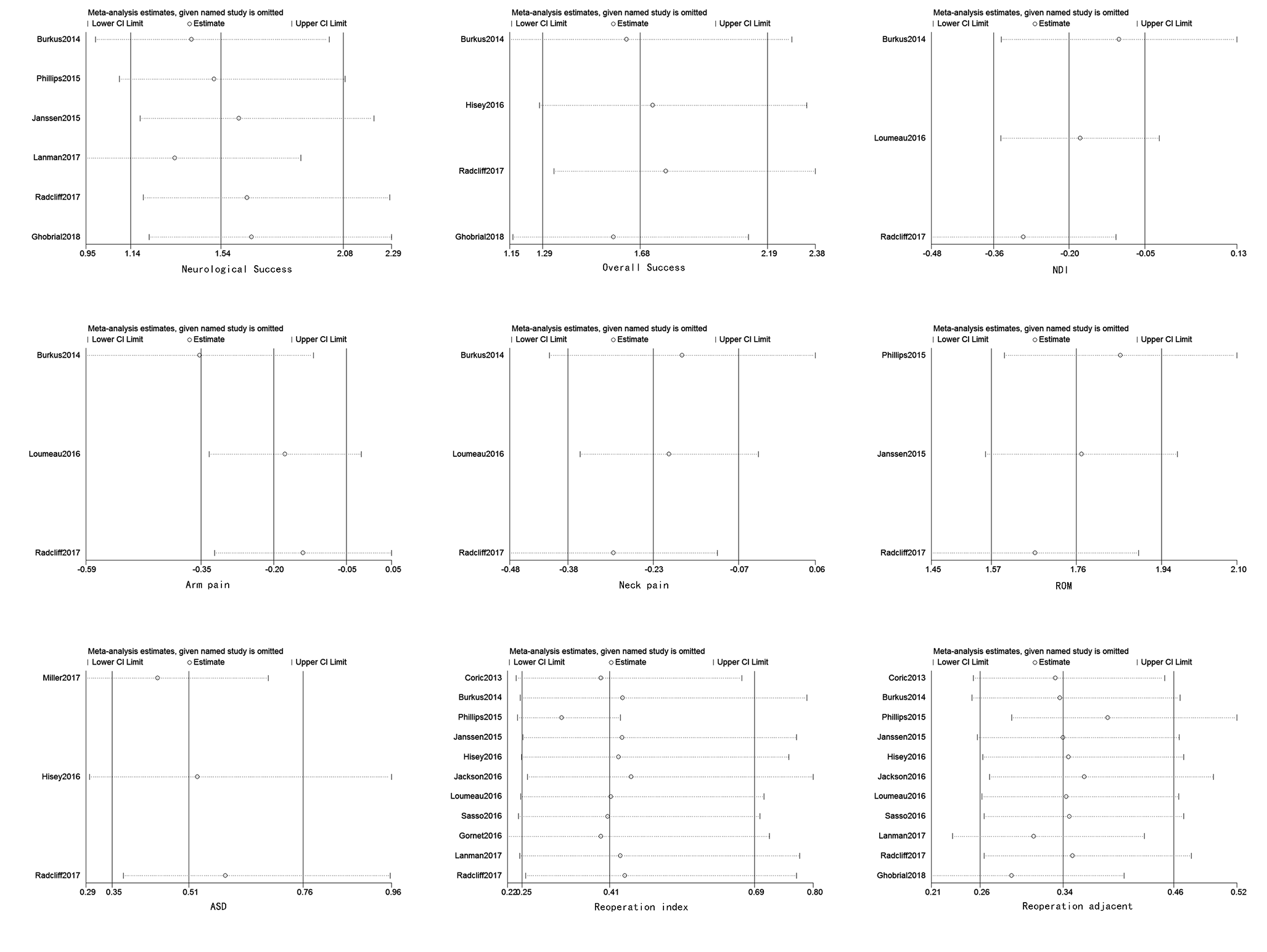

Supplement: Supplementary file 2 — Additional file 2. [file 12883_2020_1717_MOESM2_ESM.tif]
